# High-resolution grayscale image hidden in a laser beam

**DOI:** 10.1038/lsa.2017.129

**Published:** 2018-01-26

**Authors:** Fuyong Yue, Chunmei Zhang, Xiao-Fei Zang, Dandan Wen, Brian D Gerardot, Shuang Zhang, Xianzhong Chen

**Affiliations:** 1 SUPA, Institute of Photonics and Quantum Sciences, School of Engineering and Physical Sciences, Heriot-Watt University, Edinburgh EH14 4AS, UK; 2 Shanghai Key Lab of Modern Optical System, University of Shanghai for Science and Technology, Shanghai 200093, China; 3 School of Physics and Astronomy, University of Birmingham, Birmingham B15 2TT, UK

**Keywords:** grayscale image, metasurface, polarization manipulation

## Abstract

Images perceived by human eyes or recorded by cameras are usually optical patterns with spatially varying intensity or color profiles. In addition to the intensity and color, the information of an image can be encoded in a spatially varying distribution of phase or polarization state. Interestingly, such images might not be able to be directly viewed by human eyes or cameras because they may exhibit highly uniform intensity profiles. Here, we propose and experimentally demonstrate an approach to hide a high-resolution grayscale image in a square laser beam with a size of less than half a millimeter. An image with a pixel size of 300 × 300 nm is encoded into the spatially variant polarization states of the laser beam, which can be revealed after passing through a linear polarizer. This unique technology for hiding grayscale images and polarization manipulation provides new opportunities for various applications, including encryption, imaging, optical communications, quantum science and fundamental physics.

## Introduction

**Figure 1:**
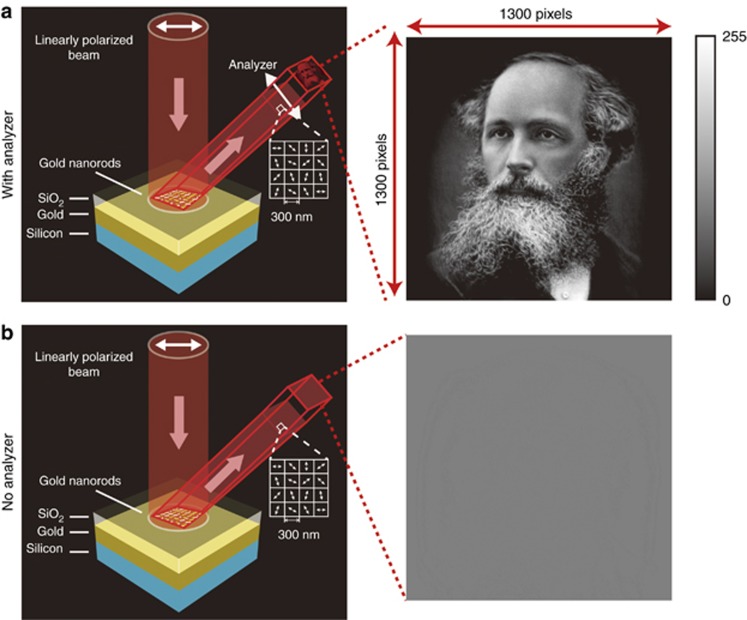
Schematic for hiding a high-resolution grayscale image. Under the illumination of linearly polarized light, two reflected beams with a spatially variant linear polarization profile are generated, which can be used to hide a high-resolution grayscale image (1300 × 1300 pixels and 256 grayscale levels). Only one reflected beam is shown here for demonstration. The two beams are exactly identical except for the propagation direction. The hidden image is revealed by an analyzer (linear polarizer) (**a**), without which no image is obtained (**b**). The metasurface consists of gold nanorods with spatially varying orientations on the top, a SiO_2_ spacer (85 nm) and a gold background layer (150 nm) on a silicon substrate. The grayscale image is encoded into the polarization profile of the resultant beam at subwavelength scale via the metasurface. The size of each pixel is 300 × 300 nm, and the overall size of the image is 390  × 390 μm.

Images consisting of optical patterns with spatially varying intensity or color profiles can be perceived by human eyes or cameras. In addition to the intensity and color, the information of an image can be encoded in a spatially varying distribution of polarization state. In this work, the first experimental demonstration of hiding a high-resolution grayscale image associated with a laser beam with a spatially inhomogeneous state of polarization is presented. Unlike optical holograms^1, 2, 3^ and recently demonstrated color images in terahertz metasurfaces^4^, for which the information is encoded in the amplitude profile of the light beam, the image here is hidden in its polarization profile. For example, holograms are recorded interference patterns of construction (intensity peaks) and destruction (elimination) of the superimposed light wavefronts. However, the images in our work are encoded in the polarization profile of the light beam with a uniform intensity distribution. Figure 1 shows the schematic of our approach for hiding an image. A grayscale image is hidden in the structured beam with a spatially variant polarization profile, which is realized by a reflective metasurface illuminated by a laser beam at normal incidence. Although only one reflected beam is shown in Figure 1 (for simplicity), two centro-symmetrically identical reflected beams are generated by the actual device. An analyzer (linear polarizer) is used to reveal the hidden image in the generated structured beam. In memory of the milestone work of James Clerk Maxwell in electromagnetics, we take one of his grayscale portraits as the hidden image. Figure 1a and 1b shows the simulation results with and without an analyzer, respectively. This approach enables us to conceal the high-capacity information in the inhomogeneous polarization profile of the laser beam and transfer the hidden information along the propagation direction of the light. This simple approach may be applied to various fields, including encryption, imaging, optical communications, quantum science and fundamental physics.

## Materials and methods

**Figure 2:**
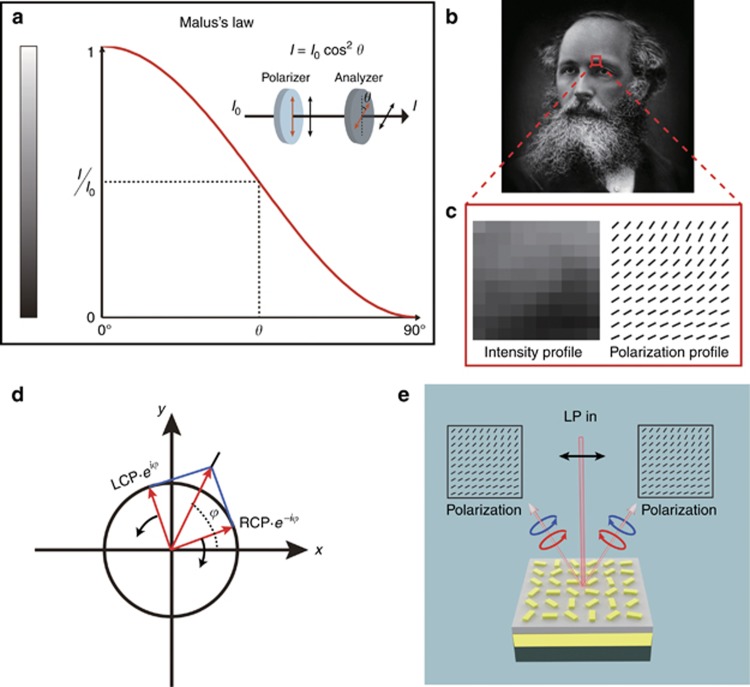
Malus’ law and image-hidden mechanism. (**a**) According to Malus’ law, when a linearly polarized light beam passes through an analyzer (linear polarizer), the intensity of light transmitted by the analyzer is *I*=*I*_0_cos^2^*θ*, where *I*_0_ is the intensity of incident light, and *θ* is the angle between the transmission axes of the analyzer and the polarizer. A grayscale image is hidden in the linear polarization profile of a light beam. (**b**) The target image of James Clerk Maxwell’s grayscale portrait. (**c**) The details of the selected area from the eyebrow area with 10×10 pixels. The left side shows the grayscale profile, and the right side shows the required polarization distribution for the analyzer with a transmission axis along the vertical direction. (**d**) A linear polarization is generated by a coherent superposition of two planar circularly polarized beams with opposite handedness, which propagates along the same direction. (**e**) As the sign of the geometric phase generated at the interface of metasurface only depends on the handedness of the incident light, under the illumination of a linearly polarized beam, the off-axis reflected beams with opposite handedness will meet, interfere with each other, and generate the desired polarization profile for the hidden image on both sides.

According to Malus’ law, when a linearly polarized light beam passes through an analyzer (linear polarizer), the intensity of the light transmitted by the analyzer is directly proportional to the square of the cosine of angle *θ* between the transmission axes of the analyzer and the polarizer (see Figure 2a), that is, *I*=*I*_0_cos^2^*θ*, where *I*_0_ is the intensity of incident light. A structured beam with inhomogeneous polarization distribution can generate a spatial intensity distribution when passing through a polarizer, which provides a new degree of freedom to encode an image. Based on Malus’ law, an arbitrary grayscale image can be hidden in the linear polarization profile of a light beam. Recently, metasurfaces^5, 6, 7, 8, 9, 10^ have enabled us to engineer the spatial distribution of the amplitude, phase and polarization response at subwavelength resolution, thus enabling us to develop a plethora of ultrathin devices with unusual properties^6, 11, 12, 13, 14, 15, 16, 17, 18, 19, 20, 21, 22, 23, 24, 25, 26, 27^.

Figure 2b shows a high-resolution grayscale image with 1300 × 1300 pixels, which is to be hidden in the optical beam. The resultant beam has a dimension of 390 × 390 μm because each pixel has a size of 300 × 300 nm, which exhibits subwavelength resolution. To explain our approach, we select an area from the eyebrow region with 10×10 pixels (Figure 2c). The enlarged intensity profile and corresponding polarization distribution are shown in the left and right sides of Figure 2c, respectively. In our design, the transmission axes of the polarizer and the analyzer (polarizer) are along the horizontal and vertical directions, respectively.

The required light beam with an inhomogeneous linear polarization profile can be decomposed into the superposition of two circularly polarized beams with equal components and opposite handedness (Figure 2d and 2e), which can be described as:


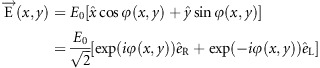


where *φ*(*x*, *y*) is the relative phase difference between two orthogonal circular polarization states; 
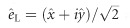
 and 
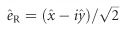
 are unit vectors of the left circular polarization (LCP) and the right circular polarization (RCP). A geometric metasurface is used to realize the handedness-dependent phase profile while maintaining a constant amplitude^10, 16, 18, 28^. Here, a single reflective metasurface is designed to generate the desired structured beams by manipulating the superposition of two beams with opposite circular polarization states, which emerge from the same metasurface (Figure 2e). The key point here is to generate a phase profile that can simultaneously generate a pair of centro-symmetrically distributed off-axis beams with the identical phase profile *φ*(*x*, *y*) upon the illumination of RCP light. As the sign of the geometric phase generated at the interface of the metasurface depends on the handedness of the incident light, when the incident beam is changed from RCP to LCP, a pair of off-axis beams with the phase profile −*φ*(*x*, *y*) is generated. Obviously, under the illumination of the linearly polarized light beam, which is the superposition of LCP and RCP components, the reflected beams with opposite handedness will combine and generate the desired polarization profile for the hidden images on both sides. A detailed explanation is provided in Supplementary Information Section 1. The combination of two sets of phase profiles for the two opposite incident handedness values will generate the desired polarization profile for the hidden image. To eliminate the effect of the non-converted beam, the off-axis configuration is used for the metasurface design. A detailed explanation of the off-axis design is also given in Supplementary Information Section 1.

## Results and discussion

**Figure 3:**
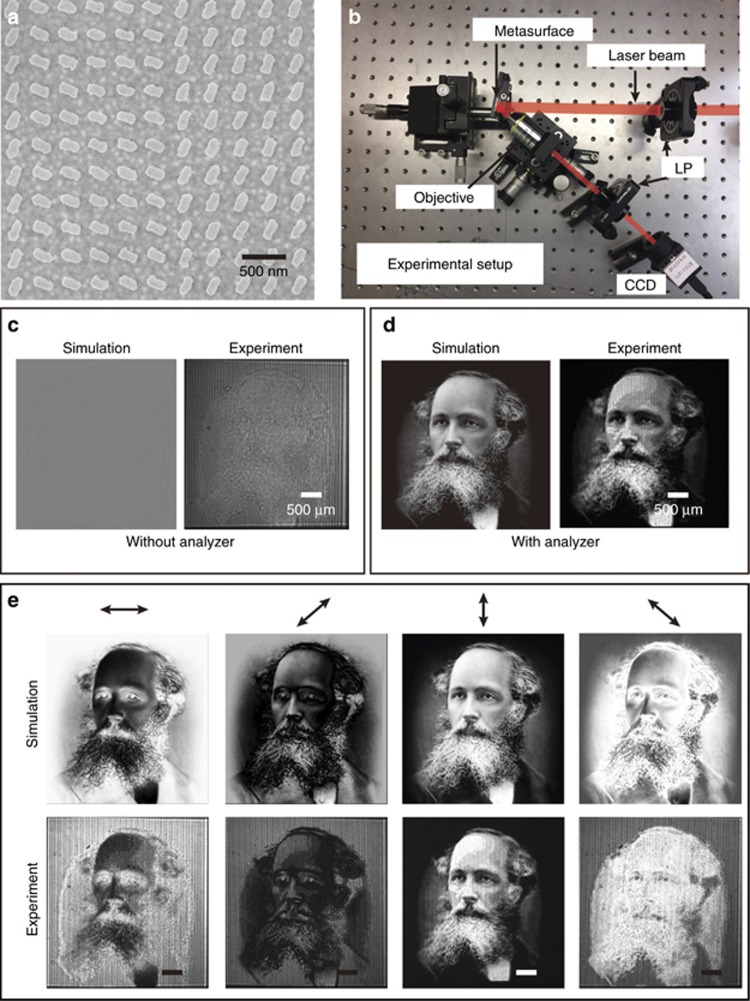
Fabricated metasurface, experiment setup and metasurface device characterization. (**a**) SEM image of the fabricated metasurface. The scale bar is 500 nm. (**b**) Experimental setup. The collimated light beam with the required linear polarization is generated using a linear polarizer (LP) and is incident on the metasurface, which is mounted on a 3D translation stage. An objective with a magnification of 10× is used to collect and expand the resultant beam. The images are captured by a CCD. The analyzer, which is a linear polarizer, is placed in front of the CCD to reveal the hidden image. The simulated and experimental results without (**c**) and with the analyzer (**d**). Note that the direction the transmission axis is along the vertical direction. (**e**) The simulated and experimental results for the analyzer with various directions of transmission axis. The results at 0°, 45°, 90° and 135° are given. The scale bar is 500 μm.

The design parameters of the metasurface and the fabrication process are presented in Supplementary Information Section 2. Figure 3a shows the scanning electron microscope (SEM) image of the part of the metasurface. To visualize the hidden image in the polarization topology of the laser beam, an analyzer (linear polarizer) is used to reveal the grayscale of the image. Thus, we do not directly observe the spatially variant polarization profile of the laser beam but indirectly confirm its existence through the intensity profile (grayscale image) behind the analyzer. For this metasurface, the additional phase difference between neighboring pixels along the *x* direction is *π*/5, where the corresponding reflection angle is 12.2° (see Supplementary Information Section 1). The experimental setup is shown in Figure 3b. An objective with a magnification of 10× was used to expand the image for visualization with a charge-coupled device (CCD) camera. Figure 3c and 3d shows the simulation and experimental results. As shown by the numerical calculation, no image is observed in the intensity profile of the beam (Figure 3c, left). The experimental result on the right side of Figure 3c confirms that the image-hidden functionality is unambiguously realized. A high-quality image is revealed (Figure 3d, right) with the analyzer whose transmission axis along the vertical direction, which is notably consistent with the simulation result (Figure 3d, left). Here, the transmission axes of the polarizer and the analyzer are along horizontal and vertical directions, respectively. The incident light beam for the simulation is a plane wave with uniform intensity, whereas the incident beam for experiment is a collimated laser beam with a Gaussian profile. The varied intensity of the incident light causes a slight discrepancy between the experimental and simulation results. Another reason for the discrepancy is the imperfection of the linear polarizer and fabrication error. Because of the off-axis design, another identical image is also observed in the reflected beam on the other side with respect to the surface normal. The clear image of the mustache, eyeball and eyebrow indicates the ultrahigh resolution of the proposed approach. To further analyze the performance of our approach, the dependence of simulated and measured results on the direction of the transmission axis of the analyzer is shown in Figure 3e. The results at 0°, 45°, 90° and 135° show the consistency between experimental and simulation results. Interestingly, the two images for the analyzer with orthogonal directions of the transmission axis (for example, 0° and 90°, 45° and 135°) are complementary grayscale images, that is, the brightest area becomes the darkest area and vice versa. The evolution process of the revealed images is clearly observed by gradually rotating the analyzer (see the video in the Supplementary Information).

To better understand the image-hidden approach, we also studied the dependence of the image on the incident polarization state and the transmission axis of the analyzer. Although our design is based on linear polarization, the device also works for elliptical polarization because an elliptically polarized light can be decomposed into LCP light and RCP light with different components. However, the image quality will be reduced. The numerically calculated and experimentally observed images hidden in the laser beam are given in different circumstances (see Supplementary Information Section 3). The measured hidden images with various combinations of the linear polarizer and the analyzer are also shown in Supplementary Information Section 3.

**Figure 4:**
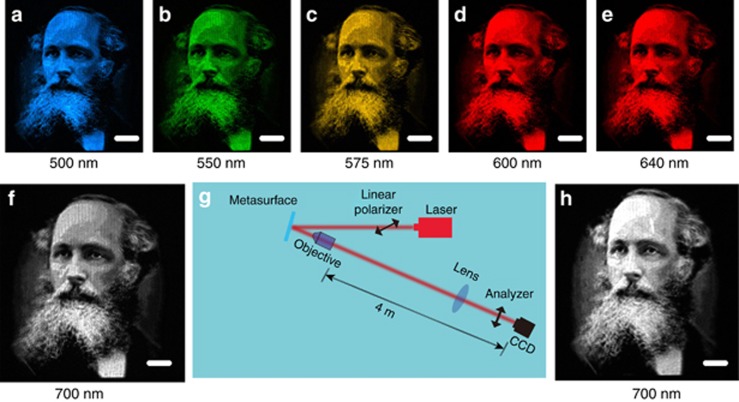
Broadband performance and robustness of the proposed approach. Experimental results at the wavelengths of (**a**) 500 nm, (**b**) 550 nm, (**c**) 575 nm, (**d**) 600 nm, (**e**) 640 nm and (**f**) 700 nm. (**g**) Experimental setup to characterize the hidden image for long-distance propagation. (**h**) Obtained image after propagating 4 m in free space. Scale bar: 500 μm. Images **a**–**e** were captured by a color CCD with 1024 × 768 pixels. Images **f** and **h** were captured by a monochrome CCD with 1280 × 1024 pixels.

Using the broadband nature of the geometric metasurface, the developed device can operate in a broad wavelength range. Images at different wavelengths were captured and shown in Figure 4a–4f. The experimentally revealed clear images at the wavelengths of 500, 550, 575, 600, 640 and 700 nm unambiguously show the operation of the developed device in the broad spectral range. The uniqueness of our approach lies in the encoding process of a high-resolution grayscale image onto the polarization profile of the laser beam. The image can be hidden and carried by a laser beam during light propagation and revealed by an analyzer. We explore the performance of the approach for long-distance propagation in free space (Figure 4g). Figure 4h shows the measured images after a 4-m propagation in free space. The image remains clearly observed, although there is a slight reduction change in image quality.

The obtained image shows how the electric field is oriented in the beam profile of the laser beam. These hidden images demonstrate the rich polarization structure that a light beam can have at subwavelength scales. The conversion efficiency is calculated by the power of two resultant off-axis beams divided by that of the incident light. The efficiency in the wavelength range of 640–960 nm is shown in Supplementary Fig. S7, and the maximum conversion efficiency is 60% at the wavelength of 820 nm (see Supplementary Information Section 4).

## Conclusions

Our approach provides a new route for hiding a high-resolution grayscale image in the polarization topology of a laser beam, which has not been reported in the literature. The uniqueness of our approach for hiding images and precise polarization manipulation and the high resolution, bandwidth, and compactness make this technology notably attractive for diverse applications, such as encryption, imaging, anti-counterfeiting, optical communications, quantum science and fundamental physics.

## Author contributions

XC, FY and SZ initiated the idea. FY, CZ and DW conducted the numerical simulations. FY fabricated the samples. FY, CZ and XZ performed the measurements. All authors discussed and analyzed the results. XC and SZ supervised the project. All authors prepared the manuscript.
