# Supplementary material for: High-resolution grayscale image hidden in a laser beam
Source: Light Sci Appl. 2018 Jan 26;7:17129–. doi: 10.1038/lsa.2017.129 (PMC6107048; doi:10.1038/lsa.2017.129)
Supplement: Supplementary Information [file lsa2017129x1.docx]

Supplementary information for

**High-resolution grayscale image hidden in a laser beam**

**Fuyong Yue^1^, Chunmei Zhang^1^, Xiao-Fei Zang^1, 2^, Dandan Wen^1^, Brian D. Gerardot^1^, Shuang Zhang^3†^, Xianzhong Chen^1*^**

1. *SUPA, Institute of Photonics and Quantum Sciences, School of Engineering and Physical Sciences, Heriot-Watt University, Edinburgh EH14 4AS, UK*
2. *Shanghai Key Lab of Modern Optical System, University of Shanghai for Science and Technology, Shanghai 200093, China*
3. *School of Physics and Astronomy, University of Birmingham, ,Birmingham B15 2TT, UK*

**†** Email: s.zhang@bham.ac.uk

* Email: x.chen@hw.ac.uk

**Section 1. Off-axis design of the metasurface and the polarization profile generation.**

Figure S1 shows the schematic to generate the required linear polarization profile. To eliminate the effect of the non-converted beam, we adopt the off-axis configuration in this design. A general linear polarization topology can be generated by a coherent superposition of two planar circularly polarized beams with opposite handedness, which propagate along the same direction. The key point here is to generate a phase profile that, upon the illumination of circularly polarized light, can simultaneously generate a pair of centro-symmetrically distributed off-axis beams with the identical phase profile with respect to the propagation axis of the incident light (see Figure S1 (i)). The required phase distribution is governed by^1^

Where is the position-dependent phase difference between the two orthogonal polarization states. $\pm\delta(x)$ is an additional phase profile, which is used to produce the phase gradient along *x* direction for the off-axis reflection ($\pm1$ order). The angle of reflection is determined by the generalized Snell’s law of reflection^2, 3^, $\sin(\alpha_{r})-\sin(\alpha_{i})=\frac{\lambda_{0}}{2\pi n_{i}}\frac{d\delta(x)}{dx}$, where $\delta_{r}$ and $\delta_{i}$are reflection and incident angle, $\lambda_{0}$ is wavelength in vacuum, $n_{i}$ is refractive index of the medium. As the sign of the geometric phase generated at the interface of metasurface depends on the handedness of the incident light, when the incident beam is changed from RCP to LCP, a pair of off-axis beams with the phase profile$-\Phi(x,y)$ are generated. Obviously, under the illumination of a linearly polarized light beam, the reflected beams with opposite handedness will meet and generate the desired polarization profile for the hidden images on both sides as shown in Figure S1.


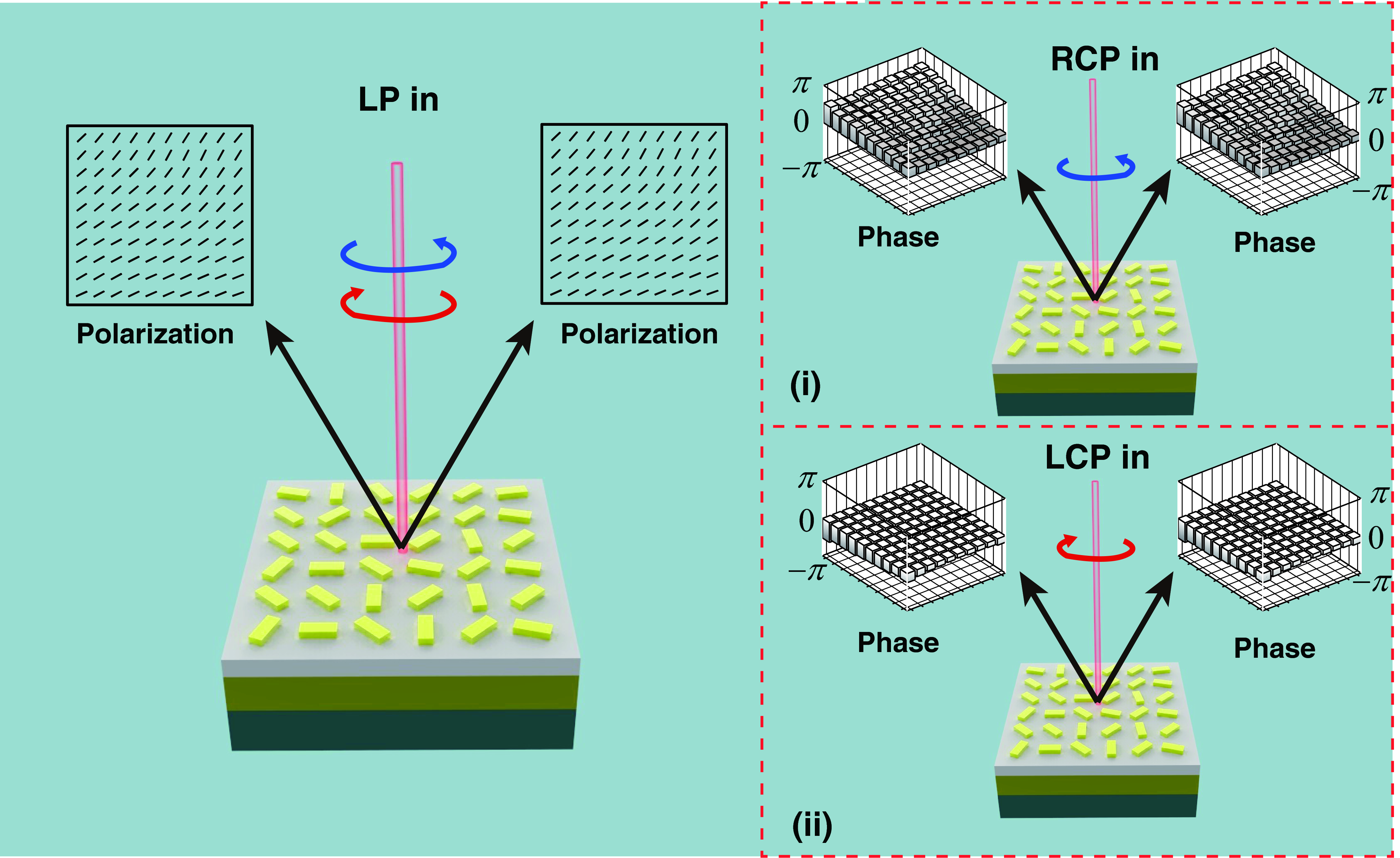


**Figure S1. Mechanism of the polarization manipulation via metasurface.** The polarization states of incident beam in (i) and (ii) are right circular polarization (RCP) and left circular polarization (LCP), respectively. A pair of off-axis beams with phase profile $\Phi(x,y)$ (or$-\Phi(x,y)$) are generated by shining the metasurface with light beam with RCP (or LCP). When a linearly polarized (LP) beam is incident on the metasurface, the reflected beam with opposite circular polarization and equal components will meet and generate the required polarization profile on both sides (Figure S1 left).

**Section 2. Fabrication procedure of the metasurface.**

The reflective metasurface is composed of three layers: a gold ground layer, a silicon dioxide (SiO_2_) spacer layer, and a top layer of gold nanorods. All the nanorods have same geometry but different orientation angles. The standard electron-beam lithography (EBL) and following lift-off process are used to fabricate the designed metasurface. First, silicon substrates are cleaned with acetone in ultrasonic bath for 10 min followed by isopropyl alcohol (IPA) for 10 min. Then the substrates are rinsed in deionized water and dried with compressed air. After that, the gold layer (150 nm) is deposited onto the silicon substrate by using the electron beam evaporator followed by the deposition of silicon dioxide (SiO_2_) layer (85 nm). The film thickness is controlled by using a calibrated film thickness monitor. The positive poly methyl methacrylate (PMMA) 950 A2 resist is spin coated on the SiO_2_ layer at 1000 rpm for 60s followed by 1500 rpm for 15 s, producing a PMMA film with a thickness of 100 nm. Then the sample is baked on a hotplate at 180 ^o^C for 5 mins. The nanopatterns are defined in the PMMA film using EBL (Raith PIONEER, 30 KV). The sample is developed in MIBK:IPA (1:3) for 45 s followed by stopper (IPA) for 45 s. A thin gold layer (30 nm) is deposited on the developed sample using electron beam evaporator. For the adhesion purpose, a thin titanium layer (3 nm) is deposited on the SiO_2_ layer prior to the gold layer. Finally, the metasurface (see Figure S2) is ready for characterization after the lift-off process in acetone.


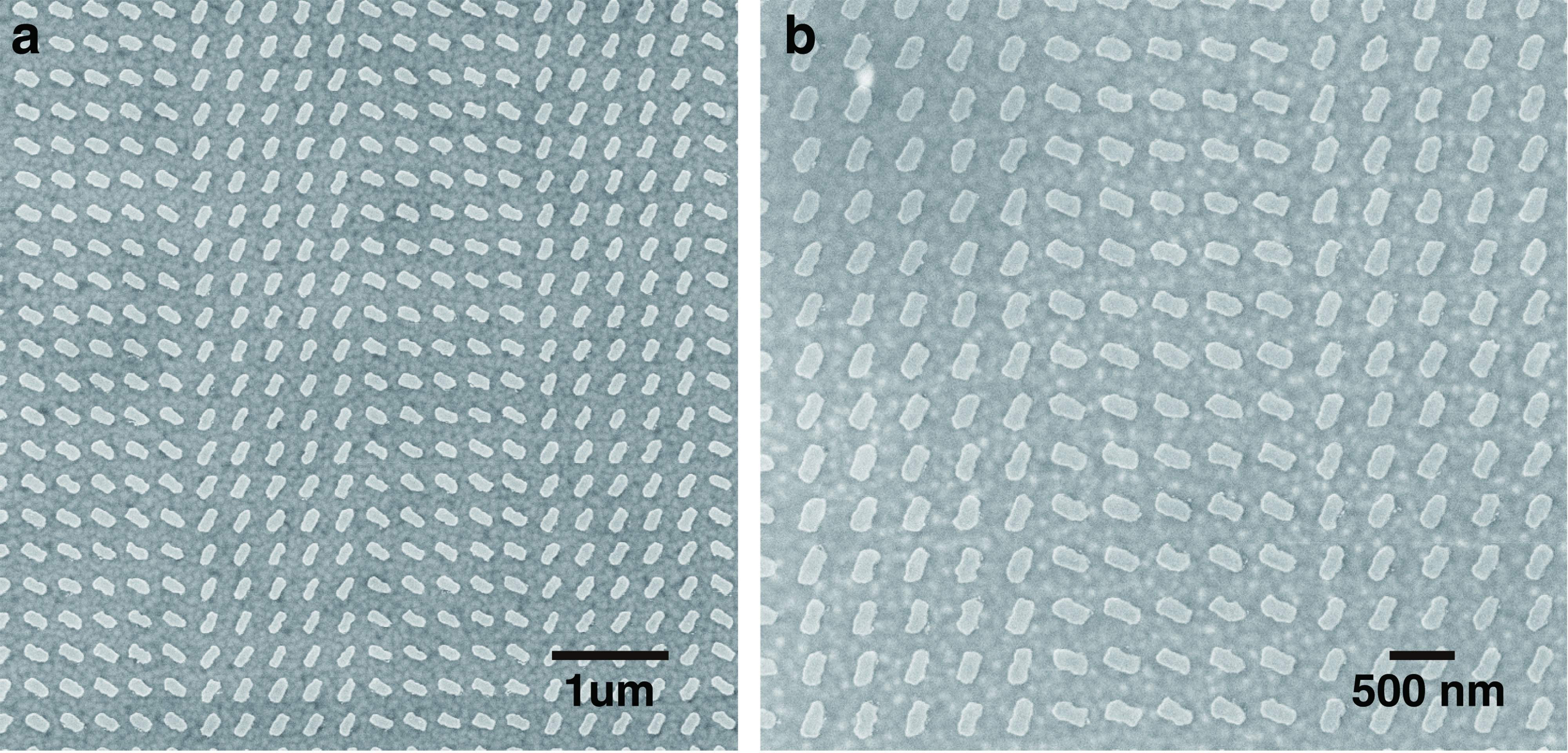


**Figure S2. Scanning electron microscope (SEM) images of the fabricated metasurface.**

**Section 3. Characterization of the developed metasurface device.**

To characterize the performance of our device at different polarization states of the incident light, the transmission axis of the analyzer is fixed along the vertical direction. The polarization state is changed by controlling the angle between the transmission axis of the polarizer and the fast axis of the quarter waveplate. These simulation and experimental results are shown in Figure S3. Although the image quality at LCP and RCP incident light is slightly reduced from that of the simulated results, the dependence of the revealed images on the polarization states of the incident light agrees well with the prediction. The minor difference for circular polarization states is mainly due to the imperfection of the experimental setup (e.g., our design is based on ideal plane wave) and fabrication error.


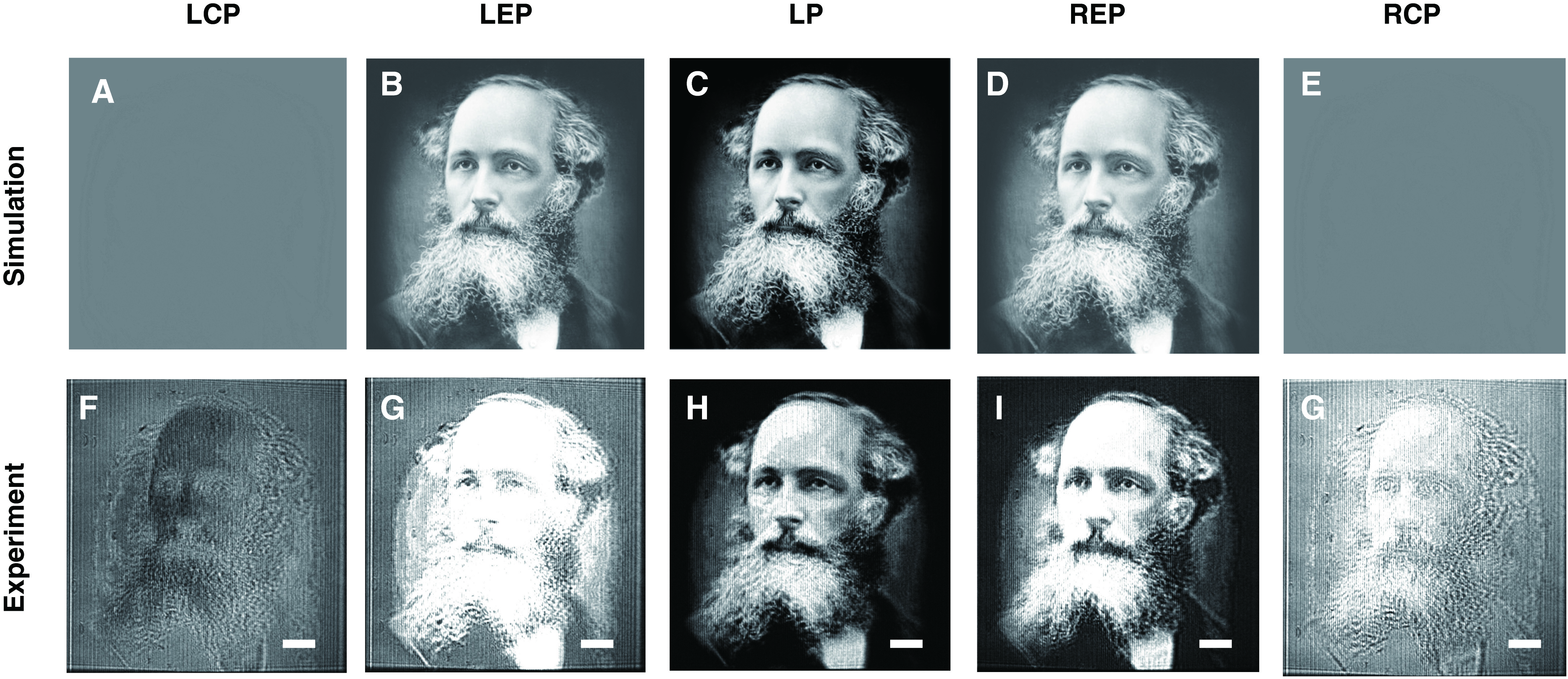


**Figure S3. Simulation and experimental results versus incident polarization states at 640 nm.** The polarization states of the incident light are chosen to be LCP, left-handed elliptically polarized (LEP), linearly polarized (horizontal) (LP), right-handed elliptically polarized (REP) and RCP. (**a-e**) Simulated images. (**f-g**) Experimental images. Scale bar, 500$\text{μm}$.

According to the Malus’ Law, the intensity of the light transmitted by an analyzer is directly proportional to the square of the cosine of angle between the transmission axes of the analyzer and the polarizer. Consequently, the quality of the hidden image (in comparison with target image) should be the same once the angle between the transmission axes of the analyzer and the polarizer is fixed. In our experiment, we set the transmission angle of the polarizer at $0^{o}$,$45^{o}$,$90^{o}$,$135^{o}$, and adjust the analyzer to make sure the transmission axis of the analyzer is perpendicular to that of the polarizer. It is worth mentioning that the best image quality is for the angle between the transmission axes of the analyzer and the polarizer fixed at 90^o^. From the simulation and experimental results shown in Figure S4, the clear images are revealed in this case. The experimentally obtained images are captured with a monochrome CCD camera at the wavelength of 640 nm.


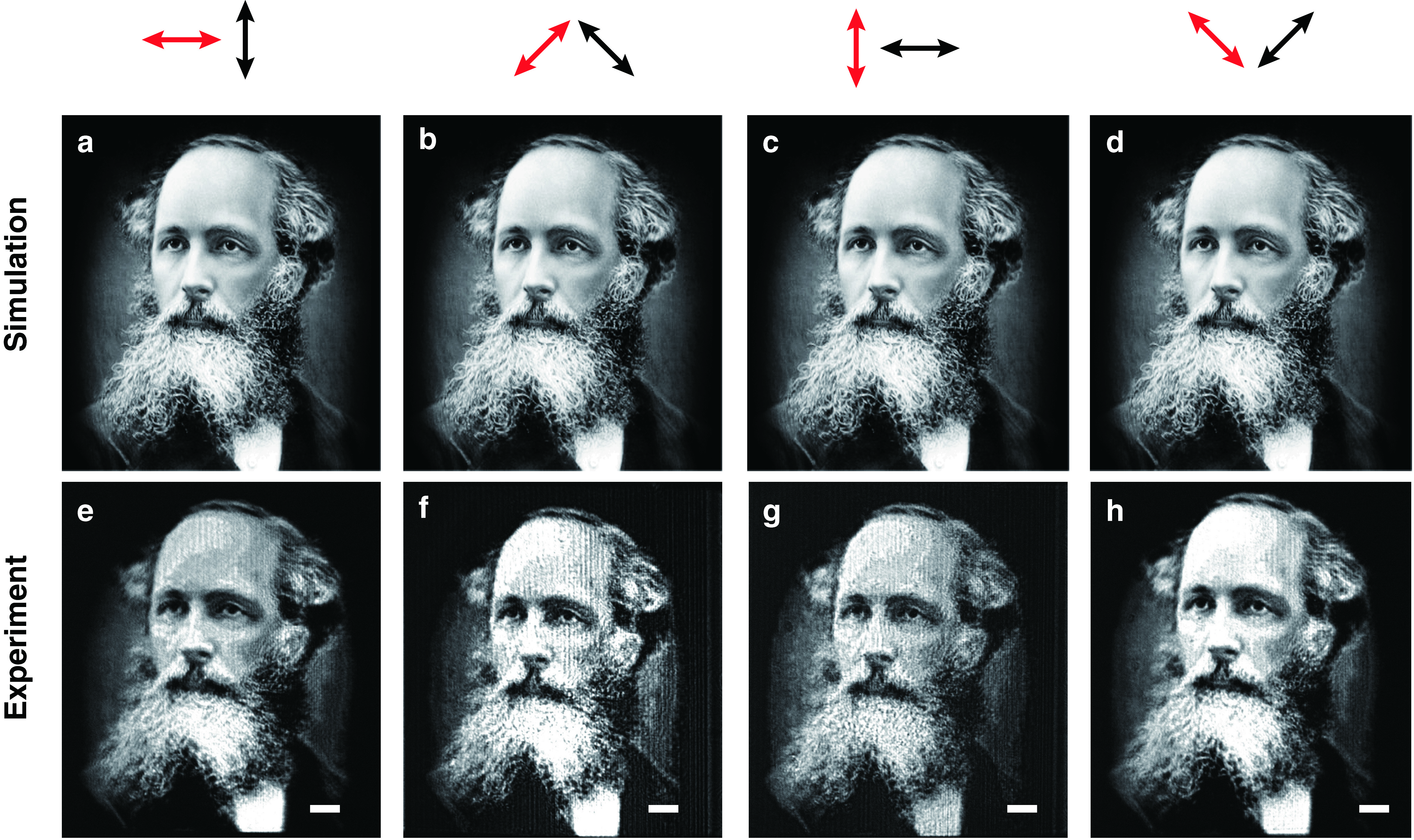


**Figure S4. Simulated and experimentally obtained images when the angle between the transmission axes of the analyzer and the polarizer is fixed at 90^o^.** The red and black double-headed arrows represent the transmission axes of the linear polarizer and the analyzer, respectively. The transmission axis of the polarizer is set at $0^{o}$,$45^{o}$,$90^{o}$,$135^{o}$ with respect to the horizontal direction, and the analyzer is adjusted to maintain the transmission axis of the analyzer perpendicular to that of the polarizer. (**a-d**) Simulated images. (**e-h**) Experimental images. Scale bar: 500$\text{μm}$.

**Section 4. High efficiency and broadband performance of the reflective metasurface.**

In metallic structures at nanoscale, oscillating free electrons at the surface of structures can be exited under illumination of a light beam due to the resonant electronic-electromagnetic oscillation. This is known as localized plasmonic resonance (LPR). The output light can be modulated by the response of the LPR. In the case of gold, the resonant frequency is at the visible wavelength range. However, the resonance occurred on gold is highly dispersive, which limits the broadband performance. We leverage on the recent progress in reflective plasmonic metasurfaces, in which a dielectric layer is sandwiched between the metallic nanostructure layer and ground metal layer. The intrinsic dispersion of the gold nanostructures is compensated by the thickness-dependent dispersion of the dielectric layer^4^. Moreover, the Fabry-Pérot effect of the multilayer structure can significantly enhance the conversion efficiency of the metasurface^5^. Assume an incident light beam $\mathbf{E}_{in}$, the total output light is a superposition of all the reflected light from the nanostructure layer and the transmitted light after propagation in the dielectric layer (see Figure S5). The output light is expressed as

$$\mathbf{E}_{out}=\mathbf{E}_{1}+\mathbf{E}_{2}+\mathbf{E}_{3}+\mathbf{E}_{4}+....$$

Now, we need to derive the complex transmission and reflection coefficients at the interface between air and the nanostructure layer $r$ and $t$, and the interface between the nanostructure and the dielectric layer $r'$ and $t'$. For simplicity of the physical model, the sheet of ultrathin nanostructures is treated as a homogeneous layer^6^.


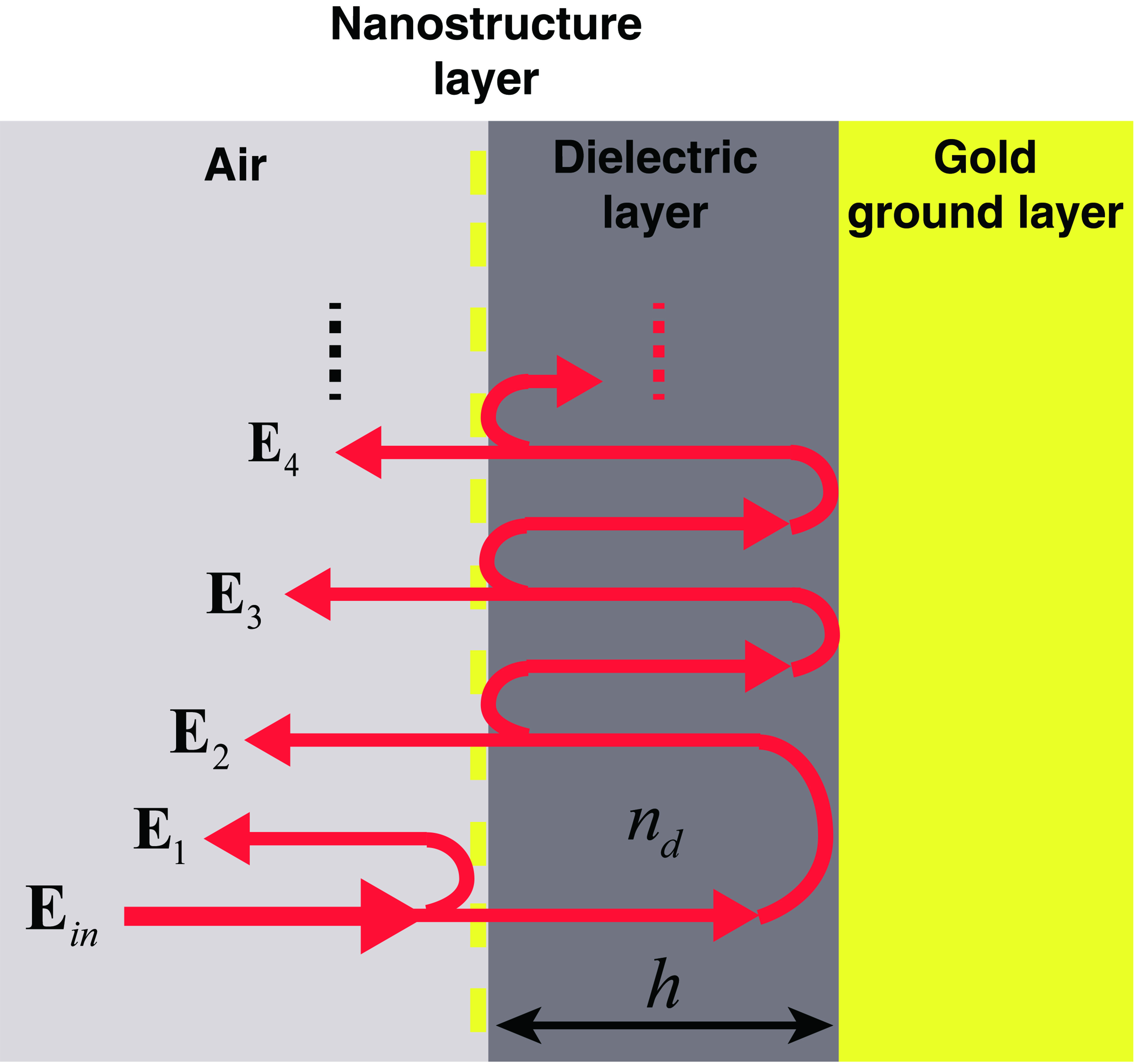


**Figure S5. Schematic of the multiple reflections from the multi-layer metasurface.**

The susceptibility of nanostructure layer is related to the polarizability of individual nanostructure as

$$\chi_{g}=\frac{\alpha_{g}}{a^{2}d}$$

where$\alpha_{g}$is the antenna polarizability, which can be assumed as$\alpha_{g}\propto\frac{1}{\omega-\omega_{0}+i\gamma}$ due to the LPR of the antennas, $d$ is the thickness of nanostructure layer. For sufficiently small$d$, the complex transmission and reflection coefficients can be approximately derived as^6^

We assume all the light is reflected on the dielectric/gold ground layer but get a phase shift . So the total output light can be given as

By properly choosing the thickness of the dielectric layer$h$, the dispersion of the complex coefficients can be cancelled by the thickness-dependent dispersion of the dielectric layer. Furthermore, the conversion efficiency can be significantly enhanced due to the Fabry-Pérot effect in the three-layer structure.

In our design, we use the silicon dioxide as the dielectric layer and simulate the efficiency of unit cell using CST microwave studio software. The Drude model is used for the gold material, where Epsilon infinity is 1, the plasma frequency is$1.37\times10^{16}$*rad/s*, and the collision efficiency is $12.15\times10^{13}$*1/s*. The length, width and thickness of the rod are 220 nm, 80 nm, and 30 nm, respectively. The thickness of gold ground layer and SiO_2_ spacer layer are 150 nm and 85 nm, respectively. The refractive index of SiO_2_ is 1.45. The simulation result is shown in Figure S6.


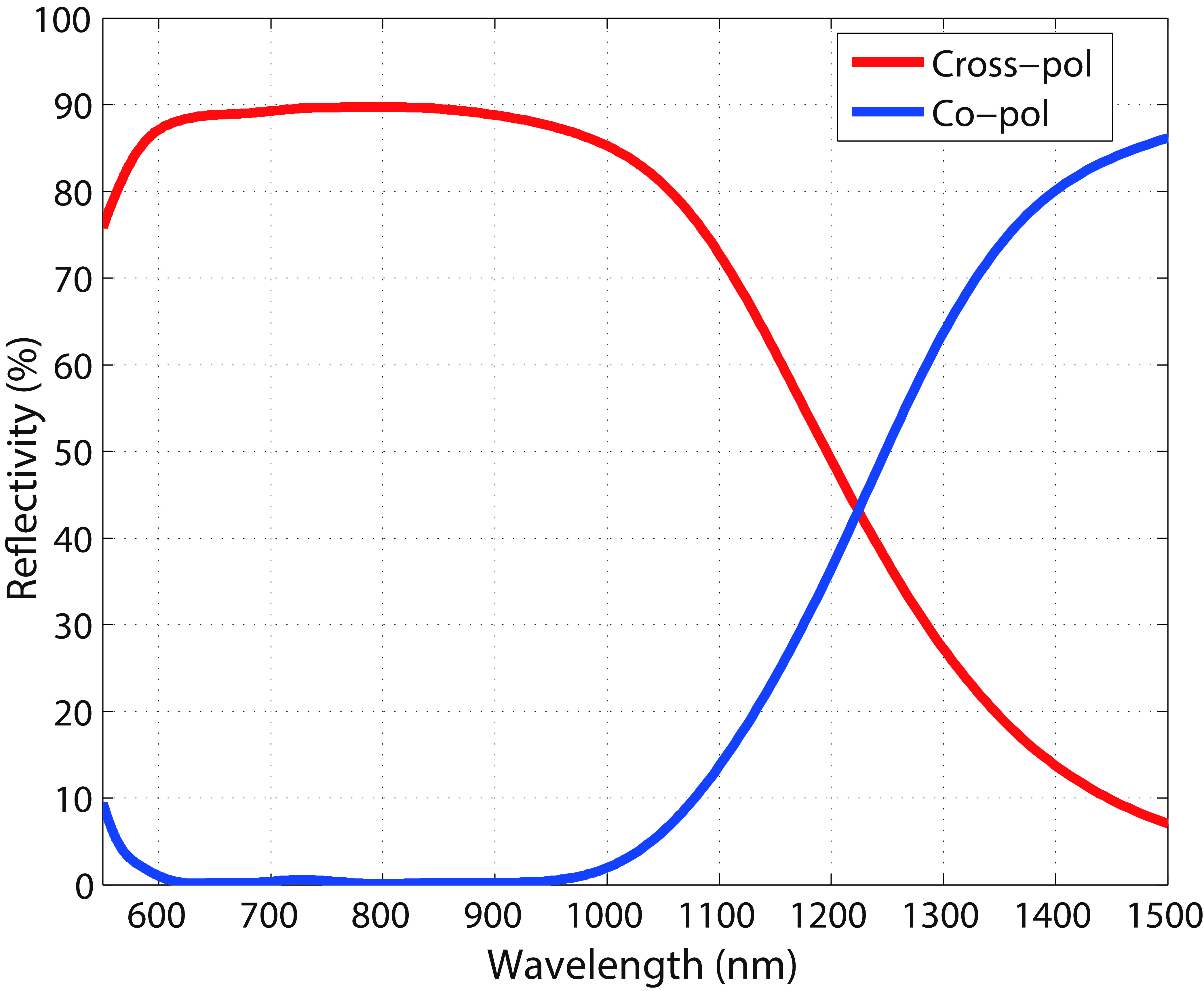


**Figure S6. Simulated conversion efficiency of single pixel of reflective metasurface.** Cross-pol and co-pol represent the converted and non-converted part of the output light, respectively.

To characterize the performance of the developed device, the metasurface is exposed to a tunable light beam from a supercontinuum laser source (NKT-SuperK EXTREME) to calculate the conversion efficiency. As described in the main text, our design is to generate the required linear polarization distribution, which is realized by the superposition of two orthogonal circularly polarized beams with certain phase profile. The two orthogonal circularly polarized beams should have the equal power and propagate exactly along the same direction. The key point is to design a phase profile that, upon the illumination of circularly polarized light, can simultaneously generate a pair of centro-symmetrically distributed off-axis beams with identical phase profile with respect to the normal axis. In our experiment, we measure the output power of two reflected off-axis beams at both sides under the illumination of right-handed circularly polarized light. The total power of two beams is normalized to unity. The relative power of two beams is shown in Figure S7 (a). We can see that the power of the two beams remain equal over a broadband wavelength range (640-960 nm). The conversion efficiency is defined by the total power of two output beams divided by the power of incident light (Figure S7 (b)). The maximum conversion efficiency is 60% at the wavelength of 820 nm. The difference between simulation and experimental results is mainly due to the titanium adhesion layer between nanopattern layer and SiO_2_ layer and the fabrication error of the nanopatterns.

**
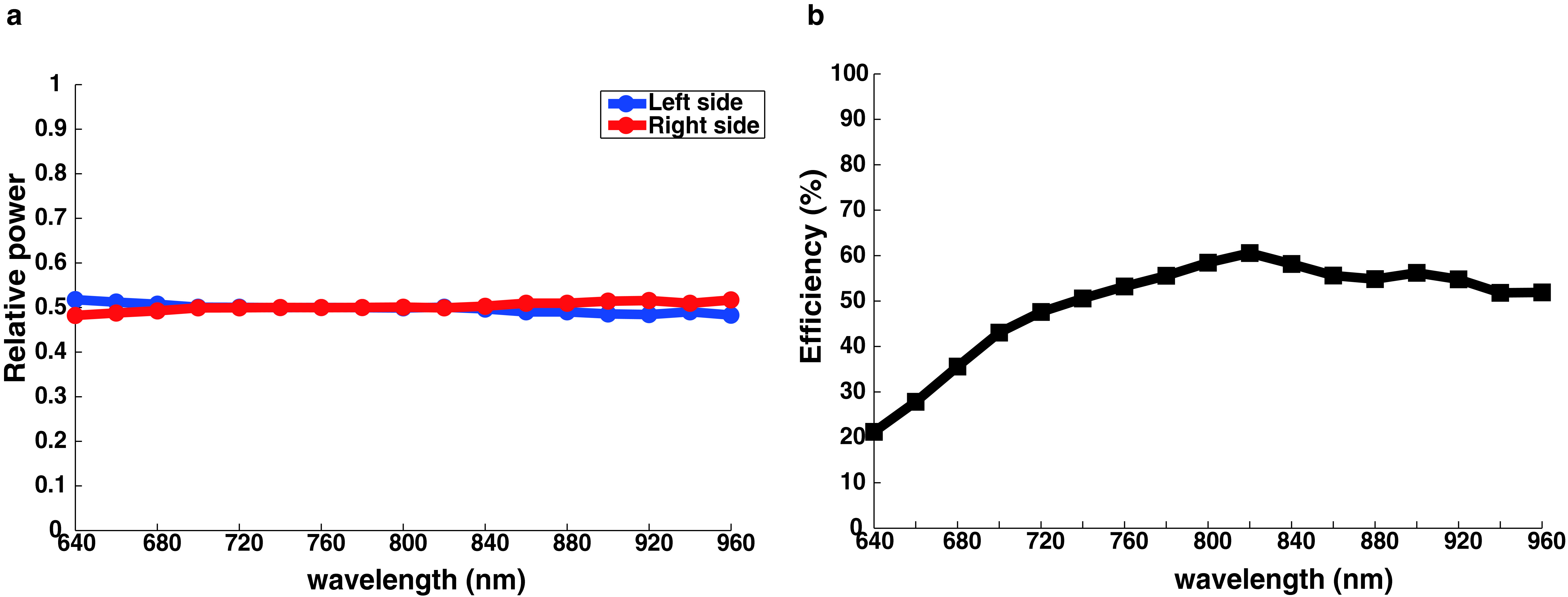
Figure S7. Relative power of two reflected beams and the conversion efficiency.** (**a**) The measured relative power of two reflected beams at two sides under the illumination of right-handed circularly polarized light. (**b**) The measured conversion efficiency is defined as the total power of the two reflected beams divided by the power of incident beam.

**Movie S1**

**Recorded video by rotating the analyzer.** This supplementary video (Movie S1) shows the evolution process of the images captured by the monochrome CCD camera by manually rotating the transmission axis of analyzer. The transmission axis of the polarizer is fixed along the horizontal direction. The continuous change of transmission axis of the analyzer is shown on the top right of the video. The experimental setup is also given on the bottom right of the video. From the video, we can see that the target image of the James Clerk Maxwell’s portrait gradually appears when the angle between the transmission axes of the polarizer and analyzer increase from$0^{o}$to$90^{o}$. While a complementary image appears when we continue increasing the angle from$90^{o}$to$180^{o}$, meaning that the brightest area will gradually become the darkest area. The observed image at$90^{o}$is the target image.

**References**

1 Yue FY, Wen DD, Zhang CM, Gerardot BD, Wang W*, et al.* Multichannel Polarization‐Controllable Superpositions of Orbital Angular Momentum States. Adv. Mater. 2017; **29** (15)**:** 1603838.

2 Yu NF, Genevet P, Kats MA, Aieta F, Tetienne JP*, et al.* Light propagation with phase discontinuities: generalized laws of reflection and refraction. Science 2011; **334**(6054)**:** 333-337.

3 Wen DD, Yue FY, Kumar S, Ma Y, Chen M*, et al.* Metasurface for characterization of the polarization state of light. Opt. Express 2015; **23**(8)**:** 10272-10281.

4 Jiang SC, Xiong X, Hu YS, Hu YH, Ma GB*, et al.* Controlling the Polarization State of Light with a Dispersion-Free Metastructure. Phys. Rev. X 2014; **4**(2)**:** 021026.

5 Grady NK, Heyes JE, Chowdhury DR, Zeng Y, Reiten MT*, et al.* Terahertz metamaterials for linear polarization conversion and anomalous refraction. Science 2013; **340**(6138)**:** 1304-1307.

6 Zheng GX, Muhlenbernd H, Kenney M, Li GX, Zentgraf T*, et al.* Metasurface holograms reaching 80% efficiency. Nat. Nanotechnol. 2015; **10**(4)**:** 308-312.
